# Lack of Cardiac Nerve Sprouting after Intramyocardial Transplantation of Bone Marrow-Derived Stem Cells in a Swine Model of Chronic Ischemic Myocardium

**DOI:** 10.1007/s12265-012-9350-2

**Published:** 2012-02-03

**Authors:** Yuan Liu, Wing-Hon Lai, Song-Yan Liao, Chung-Wah Siu, Yan-Zong Yang, Hung-Fat Tse

**Affiliations:** 1 Cardiology Division, Department of Medicine, Queen Mary Hospital, University of Hong Kong, Hong Kong, HKSAR China; 2 Research Centre of Heart, Brain, Hormone and Healthy Ageing, Li Ka Shing Faculty of Medicine, University of Hong Kong, Hong Kong, HKSAR China; 3 Department of Cardiology, First Affiliated Hospital of Dalian Medical University, Dalian, China

**Keywords:** Bone marrow cells, Ischemia, Arrhythmia, Nerve sprouting, Connecxin 43

## Abstract

Previous experimental studies suggested that mesenchymal stem cell transplantation causes cardiac nerve sprouting; however, whether bone marrow (BM)-derived mononuclear cells (MNC) and endothelial progenitor cells (EPC) can also lead to cardiac nerve sprouting and alter gap junction expression remains unclear. We investigated the effect of electroanatomical mapping-guided direct intramyocardial transplantation of BM-MNC (*n* = 8) and CD31^+^EPC (*n* = 8) compared with saline control (*n* = 8) on cardiac nerve sprouting and gap junction expression in a swine model of chronic ischemic myocardium. At 12 weeks after transplantation, the distribution and density of cardiac nerve sprouting were determined by staining of tyrosine hydroxylase (TH) and growth associated protein 43(GAP-43) and expression of connexin 43 in the targeted ischemic and remote normal myocardium. After 12 weeks, no animal developed sudden death after the transplantation. There were no significant differences in the number of cells with positive staining of TH and GAP-43 in the ischemic and normal myocardium between three groups. Furthermore, expression of connexin 43 was also similar in the ischemic and normal myocardia in each group of animals (*P* > 0.05). The results of this study demonstrated that intramyocardial BM-derived MNC or EPC transplantation in a large animal model of chronic myocardial ischemia was not associated with increased cardiac nerve sprouting over the ischemic myocardium.

## Introduction

Stem cell transplantation has been investigated as a potential novel therapy for patients with ischemic heart disease [1]. Currently, several cell types including bone marrow (BM)-derived mononuclear cells (MNC), endothelial progenitor cells (EPC), mesenchymal stem cell (MSC) and skeletal myoblasts have been tested in large animal models of chronic myocardial ischemia [2–4] and in clinical studies for cardiac repair [5–7]. Apart from clinical efficacy, a major issue with this promising therapeutic approach is the potential proarrhythmic consequences of cell therapy [8, 9].

Potential mechanisms of proarrhythmia after stem cell therapy include the heterogeneity of action potential of transplanted cells, lack of cell-to-cell connection and cardiac nerve sprouting. In experimental [10, 11] and clinical [12] studies, cardiac nerve sprouting was associated with increased incidence of ventricular tachyarrhythmia and sudden cardiac death. In large animal models of myocardial infarction [13, 14], MSC transplantation increased the magnitude of cardiac nerve sprouting. However, the effect of BM-derived MNC or EPC, which are currently the most common cell types tested in clinical trials on cardiac nerve sprouting, remains unclear. The aim of this study was to determine whether BM-MNC and BM-EPC transplantation can stimulate cardiac nerve sprouting and alter gap junction expression in chronic ischemic myocardium.

## Methods

### Porcine Model of Chronic Myocardial Ischemia

Adult minipigs (body weight of 45–50 kg) underwent ameroid constrictor (Research Instruments SW, USA) implantation around the proximal left circumflex (LCX) artery to induce chronic myocardial ischemia as described previously [4]. Animals were anesthetized with ketamine (15 mg/kg intramuscularly) and maintained by inhalation of 1.5% isoflurane. An ameroid constrictor was placed around the proximal portion of the LCX artery via a left thoracotomy to gradually induce progressive total occlusion of the artery over a 4-week period without causing acute myocardial infarction. This animal experiment was approved by the Committee on the Use of Live Animals in Teaching and Research of the University of Hong Kong.

### Study Protocol

This was a substudy of a previous animal study which investigated the functional effect of intramyocardial injection of BM-derived stem cells for treatment of chronic myocardial ischemia [4]. As described previously, at 8 weeks after ameroid constrictor implantation, the pigs with total occlusion of LCX artery and evidence of chronic myocardial ischemia as detected by left ventricular (LV) electromechanical mapping (NOGA, Biosense Webster) were selected for study. A total of 24 pigs were randomized to receive intramyocardial injection of BM-MNC (*n* = 8), BM CD31-positive EPC (*n* = 8) or saline control (*n* = 8), respectively, as guided by electromechanical mapping [4]. A total of 12–15 injections (0.1 ml per injection) were performed over the areas with hibernating myocardium as documented by electroanatomical mapping. Animals were sacrificed 12 weeks after intramyocardial injection for histological and immunohistochemical assessments to examine the in vivo effect of treatment on the ischemic myocardium. Furthermore, contrast LV angiogram was performed before and at 12 weeks after intramyocardial injection to assess LV ejection fraction.

### Preparation of Autologous BM-derived Cells

At 8 weeks after placement of the ameroid constrictor, animals were anesthetized with ketamine (15 mg/kg IM) and 60 ml of BM blood aspirated from the right iliac crest. BM-MNCs were isolated by Ficoll density gradient centrifugation [4, 5]. BM cells were washed twice in phosphate-buffered saline, resuspended in phosphate-buffered saline enriched with 10% autologous plasma to 1 × 10^7^ MNCs per milliliter and returned directly to the laboratory for use. Cell sorting of BM-EPC was performed using MACS bead selection method for CD31 (BD Pharmingen, USA) because antiswine CD34 or CD133 antibody were not available [4]. BM-EPCs were resuspended in phosphate-buffered saline enriched with 10% autologous plasma for injection. BM suspensions were tested by flow cytometry (Elite, Beckman Coulter, Fullerton, CA, USA) with directly conjugated antibodies against CD31 (BD Pharmingen, USA). Cell viability was >95% in all BM preparations.

### Immunohistochemical Staining

Cardiac nerve sprouting was studied with immunocytochemical techniques [15, 16]. Paraffin sections (4 μm) were cut from ischemic LV wall (LCX region) and septum (normal myocardium), respectively, and were then deparaffinized, rehydrated and incubated in 10% normal goat serum (Vector Labs, Burlingane, CA). Accordingly, antibodies including antigrowth-associated protein 43 (GAP-43) antibody and antityrosine hydroxylase (TH) antibody (monoclonal mouse anti-GAP-43 and anti-TH, Zymed Inc., USA. 1:250, respectively) were used for immunocytochemical staining. Furthermore, connexin 43 (monoclonal mouse anticonnexin 43 (Sigma-Aldrich, USA; 1:200) immunostaining for gap junctions was also performed [14]. Heat-induced epitope retrieval methods were used in which horseradish peroxidase was conjugated with the second antibody and then diaminobenzidine (DAB) to produce a staining pattern. Furthermore, negative controls were performed in which primary antibodies were omitted. In each animal, two random samples were taken from the ischemic area and two samples from the normal area. For each slide, five regions were randomly selected and examined. As a result, the nerve density and gap junction density were measured by the number of both GAP-43 and TH-positive cells, and connexin 43 positive cells, respectively, in a total of 10 randomly selected fields not only from the ischemic area but also from an area remote to ischemic myocardium at 400 × magnification. The results were expressed as the number of positive cells per square millimeter. All measurements were counted in a blinded fashion.

### Statistical Analysis

Data were expressed as mean ± 1 standard error of the mean (SEM). Statistical comparisons were performed with Student's *t*-test as appropriate. Comparisons between different groups were performed using one-way ANOVA followed by Bonferroni multiple comparison. Calculations were performed using SPSS software (version 15.0). *p* < 0.05 was considered statistically significant.

## Results

**Fig. 1:**
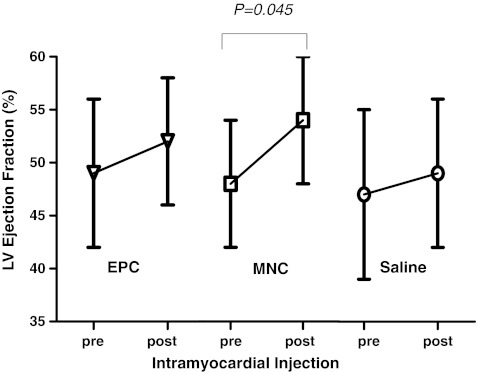
Left ventricular (LV) ejection fraction before and 12 weeks after transplantation of bone marrow mononuclear cells (BM-MNC, *n* = 8), bone marrow endothelial progenitor cells (EPC, *n* = 8) or saline control (*n* = 8), respectively

After intramyocardial injection, none of the animals with chronic myocardial ischemia developed sudden death after 12 weeks of follow-up. As determined by LV angiogram, there were no significant differences in LV ejection fraction between the three groups (48 ± 6%, versus 49 ± 10% versus 47 ± 8%, *P* > 0.05) before intramyocardial injection. At 12 weeks, transplantation of BM-MNC significantly increased LV ejection fraction as compared with before intramyocardial injection (54 ± 6%, *P* = 0.045). However, there were no significant improvements in LV ejection fraction after BM-EPC (52 ± 6%, *P* = 0.12) or saline (49 ± 10%, *P* = 0.8) injection as compared with before intramyocardial injection (Fig. 1).

**Fig. 2:**
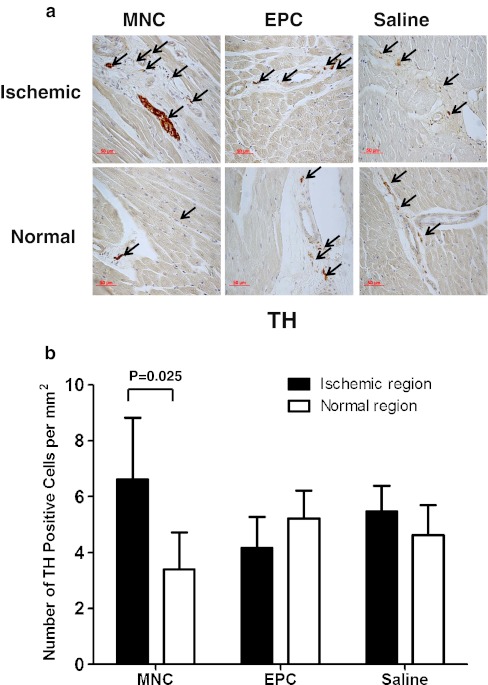
**a** Immunohistochemical staining (*arrows*) and **b** number of cells staining positive for tyrosine hydroxylase (TH) in the ischemic left ventricular wall (LCX region) and septum (normal myocardium) after transplantation of bone marrow mononuclear cells (BM-MNC, *n* = 8), bone marrow endothelial progenitor cells (EPC, *n* = 8) or saline control (*n* = 8), respectively

**Fig. 3:**
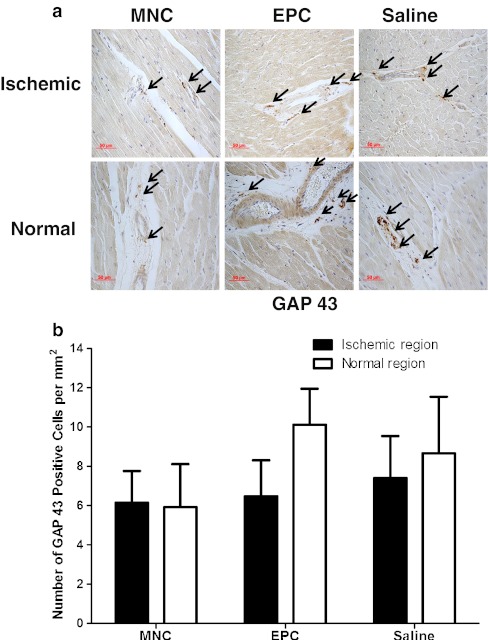
**a** Immunohistochemical staining (*arrows*) and **b** number of cells staining positive for growth-associated protein 43 (GAP-43) in the ischemic left ventricular wall (LCX region) and septum (normal myocardium) after transplantation of bone marrow mononuclear cells (BM-MNC, *n* = 8), bone marrow endothelial progenitor cells (EPC, *n* = 8) or saline control (*n* = 8), respectively

In the control group, there were no significant differences in the number of cells with positive staining of TH (5.47 ± 0.92 versus 4.63 ± 1.08 per mm^2^, *P* = 0.32) and GAP-43 (7.40 ± 2.14 versus 8.66 ± 2.88 per mm^2^, *P* = 0.68) between ischemic and normal regions, suggesting that chronic myocardial ischemia did not significantly increase cardiac nerve spouting or local sympathetic activity (Figs. 2a and 3a).

In the BM-MNC group, there was a significant increase in the number of cells with positive staining of TH (6.61 ± 2.20 versus 3.41 ± 1.31 per mm^2^, *P* = 0.025) but not GAP-43 (6.13 ± 1.62 versus 5.91 ± 2.19 per mm^2^, *P* = 0.87) over the ischemic region as compared with the normal region. In contrast, there were no significant differences in the number of cell with positive staining of TH (4.17 ± 1.11 versus 5.22 ± 0.10 per mm^2^, *P* = 0.54) and GAP-43 (6.46 ± 1.84 versus 10.09 ± 1.86 per mm^2^, *P* = 0.09) between ischemic and normal regions in the BM-EPC group. These findings suggest that BM-MNC transplantation, but not BM-EPC, increased the local sympathetic activity without nerve spouting over the ischemic myocardium.

As compared with the control group, there were no significant differences in the numbers of cells with positive staining of TH (Fig. 2b, all *P* > 0.05) and GAP-43 (Fig. 3b, all *P* > 0.05) over the ischemic and normal regions at 12 weeks after BM-MNC and BM-EPC transplantation. Similarly, there were also no differences in the number of GAP-43 and TH-positive cells over the ischemic and normal regions at 12 weeks between the BM-MNC group and the BM-EPC group (Fig. 2b, all *P* > 0.05).

**Fig. 4:**
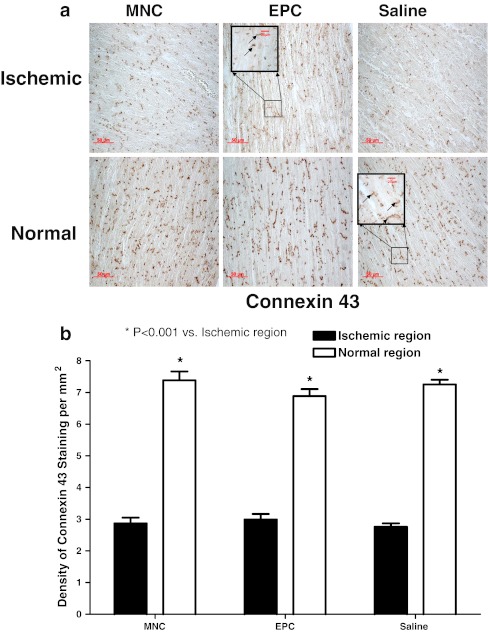
**a** Immunohistochemical staining (*arrows*) and **b** density of connexin 43 staining in the ischemic left ventricular wall (LCX region) and septum (normal myocardium) after transplantation of bone marrow mononuclear cells (BM-MNC, *n* = 8), bone marrow endothelial progenitor cells (EPC, *n* = 8) or saline control (*n* = 8), respectively

In the control, BM-MNC and BM-EPC groups, there were significant decreases in the expression of connexin 43 over the ischemic regions as compared with the normal regions (Fig. 4). As compared with the control group, there were no significant differences in the expression of connexin 43 over the ischemic and normal regions at 12 weeks after BM-MNC and BM-EPC transplantation (Fig. 4b, all *P* > 0.05). Similarly, there were also no differences in expression of connexin 43 over the ischemic and normal regions at 12 weeks between the BM-MNC group and the BM-EPC group (Fig. 4b, all *P* > 0.05).

## Discussion

Consistent with previous clinical [5–7] and experimental studies [4], our results demonstrated that transplantation of BM-MNC cells into chronic ischemic myocardium improved LV function. In contrast to previous experimental studies in animals with myocardial infarction [10, 11], we did not observe any increase in cardiac nerve sprouting in the chronic ischemic myocardium as compared to nonischemic myocardium. GAP-43 is a protein associated with axonal growth cone and is upregulated during nerve sprouting, and TH is the rate-limiting enzyme of norepinephrine synthesis, which serves as not only a marker for sympathetic nerve location but also an indirect indicator of sympathetic activity [16]. The combination of GAP-43 and TH staining can, therefore, precisely reflect the sprouting of sympathetic nerves [13, 14]. In this study, neither BM-MNC nor BM-EPC transplantation into chronic ischemic myocardium was associated with increased cardiac nerve sprouting as compared to control. Although the expression of connexin 43 was decreased in the chronic ischemic myocardium as compared with nonischemia in all three groups, BM-MNC and BM-EPC transplantation did not affect the connexin 43 expression in the chronic ischemic myocardium as compared to controls.

In large animal models of myocardial infarction [13, 14], MSC transplantation increased the magnitude of cardiac nerve sprouting. In this study, we did not observe any increase in nerve spouting after BM-MNC or BM-EPC transplantation. Although the mechanism remains unclear, the discrepancy in the results may be related to the different animal models including the species of the animals used (dogs versus farm pigs or minipigs) and the method to induce myocardial ischemia/infarction (rapid pacing versus coronary microembolization and constriction) as well as the potential difference in the propensity of stimulating nerve growth with BM-MNC and BM-EPC cells as compared with MSC. It is well known that neurons and glial cells have successfully been differentiated from MSC but not BM-MNC or BM-EPC [16]. It is possible that the paracrine effects of BM-MNC lead to increased cardiac nerve sprouting and, thus, contribute to the improvement in cardiac contractile function after transplantation [4, 17].

Furthermore, we observed that gap junction expression was not reduced significantly as determined by the density of connexin 43 staining over the chronic ischemic myocardium and that BM-MNC or BM-EPC transplantation did not alter the gap junction expression. In addition to the well known role of cell-to-cell coupling between cardiomyocytes, gap junctions also play an important role in cell-to-cell communication under various insult conditions such as hypoxia and ischemia [18]. Downregulation of the gap junctions in the ischemic myocardium is a potential protective mechanism to reduce progression of cell death during chronic ischemia [19, 20].

This study has several limitations. First, the protein levels of TH and GAP-43 were not measured in this study. Second, detailed heart rate and rhythm monitoring for the presence of spontaneous cardiac arrhythmias as well as sympathetic activity were not performed after the transplantation due to the lack of long-term telemetry monitoring system available in our laboratory. Therefore, the incidence of nonsustained ventricular arrhythmias and sympathetic activity after cell transplantation remains unclear. Third, invasive electrophysiology to assess inducible arrhythmia was also not performed. Nevertheless, we did not observe any sudden death after cell transplantation in this study. Forth, the differences in the results of this study as compared with other studies [13, 14] might also be due to the differences in the method to determine cardiac nerve spouting.

## Conclusions

In conclusion, the results of this study demonstrate that intramyocardial BM-derived MNC or EPC transplantation in a large animal model of chronic myocardial ischemia was not associated with increased cardiac nerve sprouting over the ischemic myocardium.

## Abbreviations

BM: Bone marrow
MNC: Mononuclear cells
EPC: Endothelial progenitor cells
MSC: Mesenchymal stem cell
LCX: Left circumflex
GAP-43: Growth-associated protein 43
TH: Tyrosine hydroxylase
